# Stroke rehabilitation and patients with multimorbidity: a scoping review protocol

**DOI:** 10.15256/joc.2015.5.47

**Published:** 2015-02-17

**Authors:** Michelle L.A. Nelson, Linda Kelloway, Deirdre Dawson, J. Andrew McClure, Kaileah A. McKellar, Anita Menon, Sarah Munce, Kara Ronald, Robert Teasell, Michael Wasdell, Renee F. Lyons

**Affiliations:** ^1^Bridgepoint Collaboratory for Research and Innovation, Bridgepoint Active Healthcare, Toronto, ON, Canada; ^2^Daphne Cockwell School of Nursing, Ryerson University, Toronto, ON, Canada; ^3^Ontario Stroke Network, Toronto, ON, Canada; ^4^Department of Occupational Science and Occupational Therapy, Rehabilitation Sciences Institute, University of Toronto, ON, Canada; ^5^Rotman Research Institute, Baycrest, Toronto, ON, Canada; ^6^Institute of Clinical Evaluative Sciences, London, ON, Canada; ^7^Institute of Health Policy, Management and Evaluation, University of Toronto, Toronto, ON, Canada; ^8^School of Physical & Occupational Therapy, McGill University, Montreal, QC, Canada; ^9^Toronto Rehabilitation Institute–University Health Network, Toronto, ON, Canada; ^10^College of Occupational Therapists of Ontario, Toronto, ON, Canada; ^11^St. Joseph’s Health Care, London, ON, Canada; ^12^Ontario Shores Centre for Mental Health Sciences, Whitby, ON, Canada; ^13^Dalla Lana School of Public Health, University of Toronto, Toronto, ON, Canada

**Keywords:** knowledge synthesis, scoping review, knowledge translation, stroke rehabilitation, multimorbidity, evidence-informed practice, clinical practice guidelines

## Abstract

Stroke care presents unique challenges for clinicians, as most strokes occur in the context of other medical diagnoses. An assessment of capacity for implementing “best practice” stroke care found clinicians reporting a strong need for training specific to patient/system complexity and multimorbidity. With mounting patient complexity, there is pressure to implement new models of healthcare delivery for both quality and financial sustainability. Policy makers and administrators are turning to clinical practice guidelines to support decision-making and resource allocation. Stroke rehabilitation programs across Canada are being transformed to better align with the Canadian Stroke Strategy’s Stroke Best Practice Recommendations. The recommendations provide a framework to facilitate the adoption of evidence-based best practices in stroke across the continuum of care. However, given the increasing and emerging complexity of patients with stroke in terms of multimorbidity, the evidence supporting clinical practice guidelines may not align with the current patient population. To evaluate this, electronic databases and gray literature will be searched, including published or unpublished studies of quantitative, qualitative or mixed-methods research designs. Team members will screen the literature and abstract the data. Results will present a numerical account of the amount, type, and distribution of the studies included and a thematic analysis and concept map of the results. This review represents the first attempt to map the available literature on stroke rehabilitation and multimorbidity, and identify gaps in the existing research. The results will be relevant for knowledge users concerned with stroke rehabilitation by expanding the understanding of the current evidence.

## Introduction

Stroke is a complex health event due to the wide range of associated physical and cognitive impairments. Stroke care presents challenges for clinicians, as most strokes occur in the context of other medical diagnoses. Indeed, research indicates that a stroke occurs in isolation (no co-occurring conditions) in up to 6% of patients [1, 2]. A recent assessment of organizational strengths and weaknesses for implementing best practice stroke care found that clinicians reported a strong need for education and training specific to patient/system complexity and multimorbidity [3]. An exploratory study conducted at Bridgepoint Health found that rehabilitation clinicians question the applicability of the best practices to their patients, and rely on their sound clinical judgment, teamwork, and creativity to develop treatment plans for multimorbid patients. The purpose of this scoping review is to document the extent to which multimorbidity is included in stroke rehabilitation evidence, and to identify the associated gaps in the evidence pertaining to stroke rehabilitation and multimorbidity.

Stroke is the leading cause of death and disability in Canada and globally. For those who survive, stroke becomes a chronic condition. Prevalence estimates suggest that approximately 33 million people, including 315,000 Canadians, are living with the effects of stroke [4, 5]. There are approximately 32,000 new strokes in Canada each year and 16.9 million globally [5, 6]. Highly prevalent chronic diseases (diabetes, arthritis, stroke) are known to co-occur frequently, and can co-occur with less prevalent conditions (congestive heart failure, anemia, depression) [7]. Factoring in co-occurring conditions, stroke treatment and recovery can become even more complex. Limited resources and substantial healthcare costs (acute, rehabilitation, continuing care) dictate the need to understand the factors (stroke severity, age, comorbidity, depression) that affect stroke patient outcomes and healthcare utilization [8]. Stroke patients are one of the highest users of healthcare services [9]. With mounting patient complexity [10], there is pressure to implement new models of healthcare delivery for both quality and financial sustainability.

Multimorbidity is common in stroke, with patients having on average five other chronic diseases [6, 11–13]. The precise nature and prevalence of other chronic conditions in the stroke population, however, have not been clearly reported. Evidence regarding multimorbidity and stroke has focused on the risk factors for stroke (hyperlipidemia, hypertension, diabetes, smoking, obesity) [14], with limited reporting of the prevalence of stroke plus the wider range of other chronic conditions, such as those captured by the Charlson Comorbidity Index (CCI) [15]. For the purposes of this review, *multimorbidity* is used to describe stroke rehabilitation patients with at least one other chronic condition included in the CCI [14]. Retrospective studies of stroke rehabilitation treatments have found that multimorbidity increased rates of complications, led to longer hospital stays, and was negatively correlated with functional outcomes and gains in patients – increasing the cost and decreasing the efficiency of rehabilitation [12, 16, 17]. Optimal management of multimorbidity may accelerate treatment and reduce healthcare costs for stroke rehabilitation patients [18].

Policy makers and administrators are turning to clinical practice guidelines to support decision-making and resource allocation. For example, evidence-informed care is a key component of Ontario’s Action Plan for Heath Care [19], and is posited to support the provision of high-quality, sustainable, patient-centered care. Stroke rehabilitation programs across Canada are being transformed to better align with the Canadian Stroke Strategy’s Stroke Best Practice Recommendations [20]. The recommendations provide a framework to facilitate the adoption of evidence-based best practices in stroke across the continuum of care, with the goal of transforming “stroke prevention and care by ensuring that evidence-based best practices are widely disseminated and used in the Canadian healthcare system” [20]. However, given the increasing and emerging complexity of patients with stroke in terms of multimorbidity, the evidence supporting clinical practice guidelines may not align with the current patient population as they often focus on a single condition [1].

Clinical practice guidelines are meant to assist in the provision of consistent care within a specified clinical situation, but are not necessarily expected to define a standard of care. Guidelines provide clinicians with a framework for assessing and treating clinical conditions commonly encountered in practice. Although evidence-based practice is an important tool for rehabilitation, therapy is often delivered on an individual basis, and the chronic and evolving nature of patients’ conditions, combined with the varying degrees of clinician experience, will influence the outcomes [21, 22].

Multimorbidity has been identified as one of the major challenges facing clinical practice guidelines [23]. Although quality assurance initiatives encourage concordance with evidence-based practice guidelines, there are challenges when applying guidelines developed for the treatment of single diseases in the care of patients with multiple chronic conditions [24, 25]. The high-quality evidence on which most guidelines are founded is largely based on relatively short-term randomized clinical trials (RCTs), where older age or comorbid conditions comprise the exclusion criteria [24, 26, 27]. Evidence is generally non-existent for situations where a patient suffers from several problems simultaneously [25, 28, 29]. Boyd and Fortin [30] and Fortin et al. [25] examined clinical guidelines for common chronic conditions (not stroke care) to assess the relevance to patients with multimorbidity, and found that very few practice guidelines provided any treatment recommendations for patients with two or more conditions.

The Evidence-Based Review of Stroke Rehabilitation (EBRSR) is the comprehensive research synthesis of the stroke rehabilitation intervention literature (over 2,000 studies, 1,316 RCTs) that served as the impetus and framework for changes to the Canadian national stroke rehabilitation system. The EBRSR functions as the evidence foundation for the development of the Canadian Best Practice Recommendations for Stroke Care specific to rehabilitation programs and services. However, no categorization and data extraction related to multimorbidity was conducted, recorded, or reported in the EBRSR. This is problematic for the design of stroke clinical practice recommendations. Although it may be expected (based on prevalence data) that multimorbid patients were included in reported rehabilitation intervention studies, by not having an explicit understanding of the patients included or excluded in the evidence, we may be faced with a mismatch between the participant groups used to generate evidence, the best practice recommendations, and the patient seen in practice. The purpose of this review is to understand the evidence as it relates to patients with multimorbidity. In order to do so, we will explore all evidence on stroke inpatient rehabilitation and ascertain which is applicable to patients with multimorbidity.

## Methods/Design

**Figure 1:**
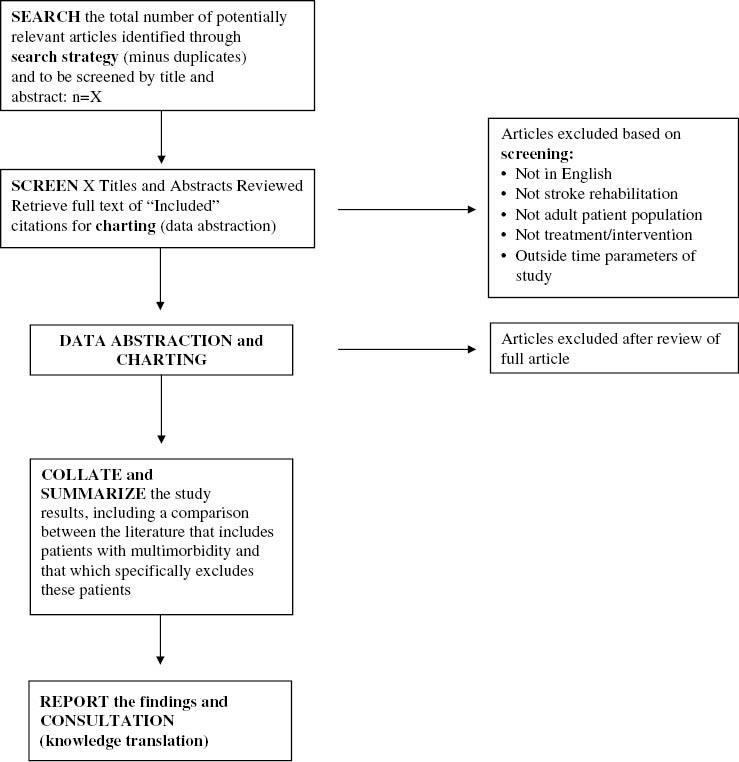
Scoping review flow diagram.

We will employ Levac et al.’s [31] advancement of Arksey and O’Malley’s [32] methodological framework for scoping reviews, implementing six iterative stages: (i) identifying the research question, (ii) identifying relevant studies, (iii) study selection, (iv) charting the data, (v) collating, summarizing, and reporting the articles, and (vi) consultation (knowledge translation). See Figure 1 for a scoping review flow diagram. Ethics Review Board approval is not required for the conduct of this study.

### Stage I: development of the research question

This research project is part of a stroke rehabilitation and multimorbidity research program led by M.L.A.N. (the principal investigator). The multisectoral team includes leaders in stroke rehabilitation research, policy, and practice, including the Ontario Stroke Network, Institute for Clinical Evaluative Sciences, the University of Toronto, Hamilton Health Region, the Evidence-Based Review of Stroke Rehabilitation, and the Bridgepoint Collaboratory for Research and Innovation. The specific research question was developed with knowledge users (Ontario Stroke Network and Toronto Stroke Network Education Coordinators) to ensure the question is aligned with the information needs of stroke rehabilitation knowledge users.

#### Research question

What is the extent and nature of stroke rehabilitation intervention evidence that includes adult patients with multimorbidity?

#### Study objectives

- To identify existing stroke rehabilitation intervention literature pertaining to patients with multimorbidity for inclusion in the Stroke Rehabilitation Evidence-Based Review and subsequently the best practice guidelines•To identify gaps in the literature and areas for future inquiry (including syntheses) that would contribute to a better understanding of stroke rehabilitation and multimorbidity interventions
- To collaborate with knowledge users to create, based on the scoping review results, an evidence map and user-informed Excel database of evidence.

### Stage II: identifying relevant studies

Using the Stroke Rehabilitation Evidence-Based Review literature search as a starting point, the team has developed and documented a framework for a search strategy and analysis of the stroke rehabilitation evidence. We have conducted a preliminary search in Ovid MEDLINE^®^ including the Medical Subject Headings (MESH) and keywords: “stroke”, or “cerebrovascular accident”, or “cerebrovascular apoplexy”, or “CVA”, or “brain vascular accident”, with “rehabilitation”, and/or “treatment” and/or “intervention”. The initial search resulted in 6,748 intervention studies, with 2,094 RCTs identified. This search will be modified and replicated in the following Ovid databases: Embase, the Allied and Complimentary Medicine Database (AMED), and PsycInfo. We will also conduct similar keyword searches in the following non-Ovid databases: Cumulative Index to Nursing and Allied Health Literature (CINAHL), Scopus, Sport Discus, and Cochrane. Reference lists and bibliographies of the articles identified by the main database searches will be searched for citations not identified by the database search.

In addition to searching the noted databases, we will conduct a thorough search of the gray literature to identify any non-indexed literature of relevance to the scoping review. The gray literature search will focus on Canadian government reports, practice guidelines, reports compiled by stroke associations, and rehabilitation organizations. Dissertation abstracts will be included in the gray literature search. Finally, other global experts in the field of stroke will be contacted and consulted in order to ensure that all relevant data are obtained. All literature searches will be conducted by the experienced Information Scientist on the study team. The studies included in the review will be amalgamated and stored using a reference management software package, ensuring there are no duplicates in the database.

### Stage III: study selection (screening)

Two reviewers will independently review and apply the selection criteria below to all abstracts, with discrepancies resolved by a third reviewer. Titles and abstracts will be screened as “include”, “exclude” or “uncertain”. Full text of articles screened as “uncertain” will be reviewed by two members of the research team and assessed against the inclusion criteria.

*Inclusion criteria*: full reports of published and unpublished studies of any quantitative, qualitative, or mixed-methods research design will be considered, including those employing comparative (e.g. randomized, controlled, cohort, quasi-experimental) methods and non-comparative (e.g. survey, narrative, audit, action-based) methods, and formative and summative evaluation reports. The scoping review will only include those studies specifically related to the topic of stroke rehabilitation interventions for adults over the age of 18 years and published between 1970 and 2013 (the time frame of the EBRSR). The inclusion of multiple research designs, and specifically non-RCTs, will enhance the representation of studies with heterogeneous samples, as comorbidity is a common RCT exclusion criterion.

*Exclusion criteria*: non-English articles will be excluded and those published before 1970.

The study inclusion criteria will be pilot tested on a convenience sample of the first 30 articles to assure a high level of inter-rater agreement. Agreement between screening reviewers will be monitored throughout in order to identify and manage “drift”.

### Stage IV: charting the evidence (data abstraction)

“Charting” describes a technique for organizing and interpreting data by sifting, categorizing, and sorting material according to key issues and themes [32]. A copy of each article/document will be obtained, individually reviewed, and charted by two reviewers. Early in the charting process, a data abstraction pilot test (approximately 30 articles) will be conducted by two research team members and results shared with the project team. Abstraction criteria will be modified if required for the full abstraction of the included studies. Data abstraction will be conducted using an abstraction form, and will be conducted by specified research team members for all articles (two researchers per article, with adjudication by a third researcher). Preliminary data abstraction elements include:

- Researcher performing data abstraction and date of data abstraction
- Identification features of the article (record number, author, year)
- Type of publication (published or unpublished)
- Study design as well as subject inclusion and exclusion criteria
- Enrollment-permitted patients with conditions listed on the CCI
- Subject characteristics (type and severity of stroke, age, sex, ethnicity, other disease characteristics, comorbid conditions)
- Type of rehabilitation intervention
- Timing of intervention
- Setting of intervention (type of facility, country)
- Type of outcomes (e.g. physical, functional, length of stay, symptoms, etc.).

### Stage V: collating, summarizing, and reporting the data

The overarching aim of this project is to scope the current evidence, summarizing the results as presented across articles, and not synthesizing or distilling specific results that would be better suited to a systematic review or a more narrow research question. As the purpose of a scoping review is to present an overview of all the information reviewed, particular attention has been paid to how the large amount of data will be summarized and presented. Three presentation strategies will be employed: (i) a modified Preferred Reporting Items for Systematic Reviews and Meta-Analysis (PRISMA) to present results from search process; (ii) a basic numerical account of the amount, type, and distribution of the studies included in the review; and (iii) a thematic analysis and concept map of the results.

**Figure 2:**
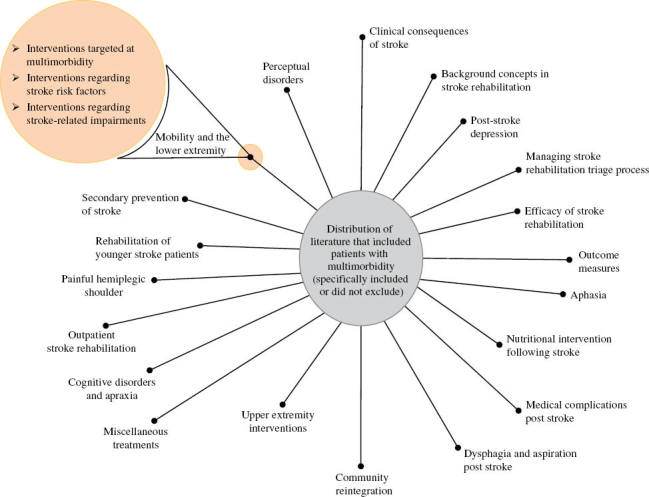
Evidence map corresponding to the content of the modules of the Evidence-Based Review of Stroke Rehabilitation.

The precise reporting format and products will be determined by study results and knowledge user requirements. We anticipate the production of tables and charts that depict the study references and counts by the following cross-tabulations: multimorbidity as it relates to study design, type of intervention, the relationship to the modules of the EBRSR, and select patient characteristics (such as age, sex, geography, ethnicity) and outcomes. An evidence-mapping approach will be used to summarize and present the results thematically; mirroring the evidence categories developed and disseminated by the EBRSR. By using the format and topic areas of the EBRSR, this evidence map will be a familiar and accessible format for knowledge users to find a summary of what evidence exists in relation to specific patient interventions and outcomes. As a companion to the evidence map, an Excel database will be developed to organize and store the evidence tabulated, again to match the organizational structure of the EBRSR synthesis. A sample evidence map corresponding to the modules within the EBRSR can be found in Figure 2.

### Stage VI: consultation (knowledge-translation) strategy

The knowledge-translation objectives for this project are: (i) to foster a partnership between stroke rehabilitation researchers and knowledge users to produce a scoping review that responds to the information needs of knowledge users, and (ii) to expedite the application of the scoping review findings into the provision of stroke rehabilitation services by key decision makers.

This project employs both integrated and end-of-grant knowledge-translation strategies, built upon an interactive process between researchers, decision makers, and knowledge users throughout all stages of the review process. The integrated knowledge-translation strategy for this project is guided by four overarching questions: (i) What are the outputs of the research? (ii) Who are the potential users of the research outputs? (iii) What are the most effective ways to make contact and interact with these users? and (iv) How do we facilitate uptake and usability of the research outputs for the appropriate audiences? [33]. Table 1 outlines key anticipated project outputs, their identified users, as well as communication strategies. To achieve an integrated knowledge-translation approach, key knowledge users have been actively engaged in the shaping of the research question, study design, and implementation plan. Knowledge users will participate in the interpretation of the results, development of presentations, and strategies for dissemination of results to ensure optimal uptake by other knowledge users. An advisory group, made up of key knowledge users, will also be established to provide insight and feedback regarding the project design and implementation, as well as for knowledge transfer. This advisory committee will be comprised of researchers, policy makers, healthcare decision makers, front-line clinicians, stroke rehabilitation experts, and the core project team. Three advisory group meetings will be held throughout the review process.

The end-of-grant knowledge-translation activities will transmit the review findings to ensure that this knowledge is made available to those who need it, and that it is packaged in a manner that is acceptable and relevant to the end users for sustained knowledge translation. This stage will include working with decision makers and knowledge users in community and inpatient rehabilitation settings to package the review findings in the most suitable way for practical and sustained application, and to determine if the recommended practices have the potential to bring about change. The following end-of-grant knowledge-translation activities are proposed; however, additional activities will be developed based on the study findings: (i) conduct a virtual (tele/video) conference meeting with key stakeholders share finding, and develop consensus on recommendations; (ii) create an evidence map as well as a web-based tabulated database of studies corresponding to the evidence categories used in the Stroke Rehabilitation Evidence-Based Review; (iii) produce a “lay language” summary report of key findings and recommendations for distribution to national and provincial organizations, including organizations that provide care services to patients with multimorbidity; and (iv) present review findings at conferences and in peer-reviewed publications.

### Anticipated challenges

The methods outlined will require keen attention to required resources and activities. Knowledge-user team members have committed organizational resources to support the review and, most importantly, the dissemination of the study findings. The most substantial threat to the successful completion of this project is the quantity of included studies. Our interim search results of MEDLINE^®^ returned up to 6,700 intervention articles that would need to be screened for inclusion in the study. With the addition of non-duplicate citations found through our other database searches and the gray literature, the number of citations could increase exponentially. To address this threat, we will likely need to refine our review focus to ensure it is feasible (given time and resources), but that it continues to produce results and products of value for the knowledge users first and foremost. We will conduct the search strategy as identified in the methods section, which will give us and the knowledge users a full understanding of the literature (all stroke rehabilitation interventions, all disciplines since 1970). This is an important knowledge product in its own right, and will serve to strengthen the existing Stroke Rehabilitation Evidence-Based Review. Prior to the screening and charting phases, the team will meet with their advisory committee members to determine the “first slice” of the literature to review. Possible strategies to reduce the number of citations may involve limiting the scoping review by health disciplines or intervention type. A second strategy may be limiting the time frame of the search, or by scoping the most recent literature first. In addition, we anticipate developing other funding applications to stakeholder organizations to scope all of the relevant literature.

Given the human resources required for this project, we will realistically be able to chart a maximum of 3,000 articles. This parameter will serve as an important consideration when determining what literature to focus our screening/abstraction on for this review. We will rely on the expertise of the project team and advisory committee members to ensure that the review methods produce results and products of value to the knowledge users.

## Discussion

The results from this proposed scoping review will be useful for researchers, clinicians, and knowledge users concerned with stroke rehabilitation, as they will identify and classify the evidence pertaining to stroke rehabilitation and multimorbid patients – and ascertain where further research is needed. Specific added value outcomes of this project (provincial and national) are anticipated at the policy level, for clinical practice, and in research.

### Policy level impact

The results from the systematic search will enhance the content of the EBRSR. This enhancement will have a direct impact on the Best Practice Recommendations, as the EBRSR is a key evidence source utilized by national decision makers in the development of the recommendations. Our key knowledge user team members have committed to informing the appropriate government offices of our study findings to support the transformation of rehabilitation services. Other national partners will support the knowledge translation of project findings across Canada through their provincial networks.

### Clinical level impact

The creation of the evidence map and database will support rehabilitation clinicians in the use of best practice recommendations by determining and documenting which current evidence is relevant for which types of patients seen in clinical practice. Findings of this study may also help knowledge users to develop broad “clinical principles” to guide rehabilitation services for patients with multimorbidity, similar to the work conducted by the American Geriatrics Society regarding patients with multimorbidity [24].

### Research outcomes

The study results will support a multidisciplinary and multisectoral research agenda in multimorbidity and stroke rehabilitation through (i) identifying research gaps in the stroke rehabilitation literature, (ii) determining where syntheses regarding stroke rehabilitation and multimorbidity are appropriate and necessary, and (iii) instigating a discussion with researchers and funding partners about the possible disconnect between the generation of “gold standard” RCT evidence and the reality of clinical practice.

Although this study is designed to assess the stroke rehabilitation intervention evidence specifically, the project methodology and study findings will be relevant to all clinical practices or programs that provide clinical care to patients with multimorbidity (e.g. geriatrics, primary healthcare, cancer, diabetes).

## Conclusion

This review draws on the expertise of an interdisciplinary team to map the available literature on stroke rehabilitation and multimorbidity, and identify gaps in the existing research. The results will be relevant for researchers, clinicians, decision makers and healthcare consumers concerned with stroke rehabilitation by expanding the understanding of the current evidence, and supporting the conceptualization of best practice guidelines for stroke rehabilitation for patients with multimorbidity. The findings of this research will also contribute to a broader program of research designed to develop and test strategies for improved rehabilitative care for patients with multimorbidity.

## Figures and Tables

**Table 1:** Knowledge-translation strategy, products, and audience.

What are the outputs of the research?	Who are the potential users of the research outputs?	What are the most effective ways to interact with these users?	How do we facilitate uptake and usability of the research outputs for appropriate audiences?
**Enhancement** of the EBRSR through a formal, systematic literature search strategy	EBRSR, Canadian Stroke System, Heart and Stroke Foundation, researchers	Collaboration with the knowledge users in the development of the research question	1. Utilize integrated knowledge-translation approaches to ensure usability of the output 2. Share the search terms and results
**Methodological approach** to map available evidence for relevance to patients with multimorbidity	This methodological contribution will be relevant to clinical areas/patient populations (e.g. geriatrics, spinal cord injuries, diabetes), researchers conducting systematic reviews	Engaging research colleagues in the project design and implementation	Consult researchers in other clinical areas to ensure approach and results are translatable to their clinical contexts and populations
**Synthesis** of the current stroke rehabilitation evidence that is relevant to the multimorbid patient population	Knowledge users (Ontario Stroke Network and regional affiliates), clinical service administrators, clinicians	Engaging research and knowledge users, team members, and colleagues in the project design and implementation	Team members and key stakeholders assist in the development of knowledge-translation materials appropriate for the different audiences
**Recommendations** regarding primary research (topic and methodological approaches) for stroke rehabilitation and patients with multimorbidity	Researchers in rehabilitation as well as other clinical programs that serve patients with multimorbidity, funding agencies	End of grant strategies (web or teleconference, presentations, publications) that discuss knowledge gaps	Engage with the researchers, funders (integrated knowledge translation) in the development of end-of-grant knowledge-translation strategies and products
**“Evidence map”** – An easy-to-manage evidence map of the evidence (type, quantity) relevant to what types of patients in what context	Knowledge users (Ontario Stroke Network, Toronto Stroke Networks, Canadian Stroke System), EBRSR, clinicians	By engaging the knowledge users in all phases of the study, including knowledge translation	This evidence map can be formatted to align with the modules of the EBRSR or the tabs contained within the StrokEngine

EBRSR, Evidence-Based Review of Stroke Rehabilitation.
